# Delirium Accompanied by Cholinergic Deficiency and Organ Failure in a 73-Year-Old Critically Ill Patient: Physostigmine as a Therapeutic Option

**DOI:** 10.1155/2015/793015

**Published:** 2015-10-13

**Authors:** Benedikt Zujalovic, Eberhard Barth

**Affiliations:** Sektion Operative Intensivmedizin, Klinik für Anästhesiologie, Universitätsklinikum Ulm, Albert-Einstein-Allee 23, 89070 Ulm, Germany

## Abstract

Delirium is a common problem in ICU patients, resulting in prolonged ICU stay and increased mortality. A cholinergic deficiency in the central nervous system is supposed to be a relevant pathophysiologic process in delirium. Acetylcholine is a major transmitter of the parasympathetic nervous system influencing several organs (e.g., heart and kidneys) and the inflammatory response too. This perception might explain that delirium is not an individual symptom, but rather a part of a symptom complex with various disorders of the whole organism. The cholinergic deficiency could not be quantified up to now. Using the possibility of bedside determination of the acetylcholinesterase activity (AChE activity), we assumed to objectify the cholinergic homeostasis within minutes. As reported here, the postoperative delirium was accompanied by a massive hemodynamic and renal deterioration of unclear genesis. We identified the altered AChE activity as a plausible pathophysiological mechanism. The pharmacological intervention with the indirect parasympathomimetic physostigmine led to a quick and lasting improvement of the patient's cognitive, hemodynamic, and renal status. In summary, severe delirium is not always an attendant phenomenon of critical illness. It might be causal for multiple organ deterioration if it is based on cholinergic deficiency and has to be treated at his pathophysiological roots whenever possible.

## 1. Introduction

Postoperative delirium is a common problem in intensive care unit (ICU) patients [1]. The etiology is based on multifactorial clinical disorders, for example, age, medication, and trauma/infection, and therefore, a causal therapy remains often difficult. The cholinergic deficiency and thus neurotransmitter disbalance are thought to be a final pathway in the pathophysiology of delirium [2].

Nitsch et al. suggested that AChE gene is activated by cholinergic neurotransmission via a feedback mechanism, leading to increased formation of AChE protein and accelerated degradation of acetylcholine at cholinergic synapses [3]. Based on this, we assumed that high AChE activity leads to a fast degradation also of high concentration of acetylcholine in the synaptic cleft which consecutively results in a cholinergic deficiency. Up to now, directly detecting the cholinergic deficiency is not possible. Additionally, the postsynaptic acetylcholinesterase is membrane bound. However, the determination of the erythrocytic acetylcholinesterase activity (AChE activity) as a surrogate marker of the cholinergic transmitter homeostasis is feasible. As mentioned by Flacker et al., an elevated serum anticholinergic activity is independently associated with delirium [4].

The indirect parasympathomimetic physostigmine allows a pharmacological approach to readjust cholinergic homeostasis after detection of AChE activity derangement.

In this paper, we report the successful therapy of a 73-year-old male delirious ICU patient accompanied by deteriorated hemodynamic and renal function.

## 2. Case Presentation

**Figure 1:**
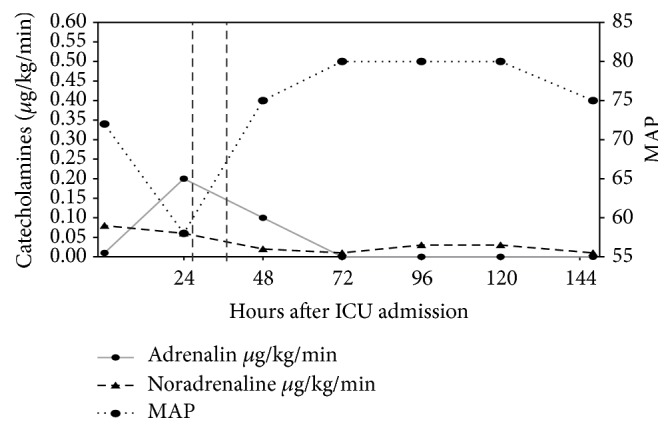
Time course of adrenaline and noradrenaline demand (*μ*g/kg/min) in relation to the physostigmine application (highlighted with vertical lines). Resulting mean arterial blood pressure (MAP) is presented with the dashed line.

The 73-year-old male patient was admitted postoperatively to the ICU. He received a cardiac resynchronization therapy (CRT) system with epicardial left-ventricular electrode via minithoracotomy due to severe dilatative cardiomyopathy. At the time of admission, he was mechanically ventilated and deeply sedated with propofol (Richmond Agitation Sedation Scale (RASS) −5). Analgesia was provided with continuous infusion of sufentanil. The patient received low doses of adrenaline and noradrenaline. After meeting criteria, he was extubated 3 hours after ICU admission. 4 hours later, the patient revealed hemodynamic deterioration with hypotension and consequently massive enhanced catecholamine demand (Figure 1).

**Figure 2:**
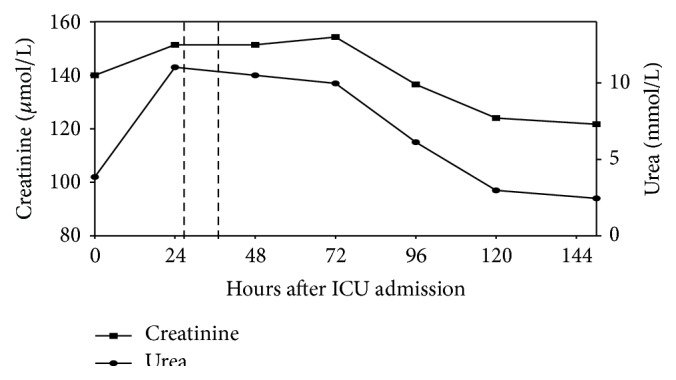
Parameters of renal function over the ICU stay. Administration of physostigmine is highlighted with vertical lines.

There had been neither signs of hemorrhage nor signs of edema in the performed chest X-ray and ultrasound of the heart and lungs. We implemented an advanced hemodynamic monitoring using the PICCO system. The heart index was 3.3 L/min under the proceeded catecholamine therapy. Additionally, the patient developed an acute renal failure with oliguria and hyperkalemia (in maximum 5.8 mmol/L) (Figure 2).

Furthermore, the patient, despite being awake, did not react to prompts or was not able to speak. He was hypoactive delirious as detected by CAM-ICU (confusion assessment method for the ICU). The RASS score at this time was −2. There was no evidence for a hallucinatory or an anxious component. A similar episode in the past was denied by the patient's relatives. We checked the pre- and perioperative medication by using the anticholinergic-cognitive-burden-scale (an overview of potential and evidentially anticholinergic drugs). We had no evidence for a central anticholinergic syndrome (no atropine and no neuroleptics were used in the perioperative setting, and no fever, agitation, and hyperactivity could be detected) [5].

Despite all the efforts, this status with catecholamine refractory hypotension in spite of the adequate volume resuscitation (mean central venous pressure was 22 mbar) and hypoactive delirium was going on for nearly 24 hours. Due to this situation, we decided to determine the acetylcholinesterase activity (AChE activity) using the on-site device ChE checkmobile, which is an in vitro diagnostic tool for the determination of cholinesterase activity using whole blood. The ChE checkmobile was originally developed for the German armed forces for the detection of acute poisoning (e.g., pesticides and others) [6].

**Figure 3:**
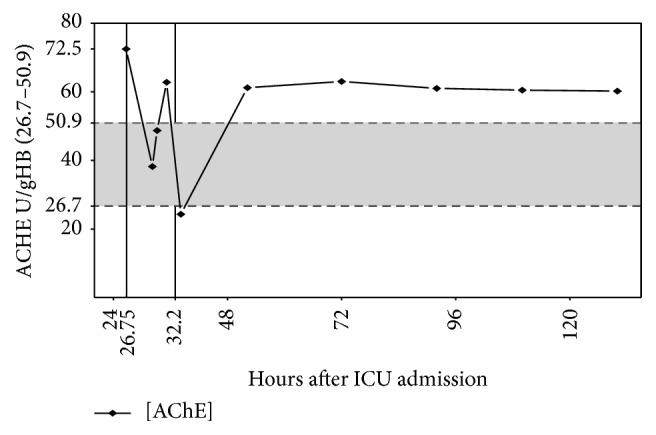
Levels of AChE activity. Moments of physostigmine application (hours after admission to the ICU) are highlighted with vertical lines. The shadow indicates the reference area of AchE activity.

The patient's AChE activity of 72.50 U/gHb was high above the reference range (26.7–50.9 U/gHb). As a consequence, we administered 2 mg physostigmine over 30 minutes (added to 100 mL NaCl 0.9%) (Figure 3). Ten minutes after application of the initial physostigmine dose, the patient's vigilance increased rapidly (from RASS −2 to RASS +1). The CAM-ICU became negative; that is, the patient had no longer a delirium. Concurrently, we observed a hemodynamic stabilization with decreased catecholamine demand (Figure 1) followed by an improvement of renal function within the next hours (Figure 2). A second dose of 2 mg physostigmine was administered preemptively 8.5 hours after the first dose because of the reincreasing AChE activity. On day 5 after ICU admission, the patient could be relocated to the intermediate care unit of his local hospital with a persistent negative CAM-ICU and normalized hemodynamic and renal function during the last 4 days.

## 3. Discussion

In this case report, the elevated AChE activity could be interpreted as a sign of an increased substrate depletion of acetylcholine in the synaptic cleft. Up to now, it is unknown which AChE activities are physiologic under which condition. Actually, it is part of the prospective, multicenter observational study CESARO to define acetylcholine esterase activities in patients in the perioperative setting. Hitherto, the given reference range of AChE activity should be considered as a guidance level.

The function of the central nervous system is dependent on several neurotransmitter systems and their interaction. One of these is the cholinergic system, whose main transmitter is acetylcholine. Acetylcholine is important for cortical arousal, attention, learning, and memories. Anticholinergic influence can cause a delirium, even in healthy volunteers [7]. In our report, we mentioned the influence of cholinergic activity on several organ systems. The initial massive enhanced AChE activity and the delirium and other organ failures represent a complex of pathophysiological symptoms. As described in several studies in rodents, ventricular dysfunction could be improved by administering the acetylcholinesterase inhibitor pyridostigmine [8]. Acetylcholine, if it adheres to the renal endothelium, leads to vasodilation by stimulating the endothelial NO synthase (eNOS) which results in NO release [9]. Thus, the amount of renal perfusion is influenced by cholinergic activity. It is conceivable that we improved the renal function during the high-dose catecholamine therapy because of the increased acetylcholine level after physostigmine application. Another notable aspect is that stimulating the vagus nerve leads to an acetylcholine increase. Acetylcholine significantly attenuates the release of proinflammatory cytokines (TNF, IL-1*β*, IL-6, and IL-18) but not that of the anti-inflammatory cytokine Il-10 [10]. The surgical procedure in combination with the chronic heart disease contents a higher risk for developing SIRS/sepsis. Improving the cholinergic transmission using the indirect parasympathomimetic physostigmine could mitigate the expected inflammatory response too.

In summary, measuring the AChE activity in ICU patients with indistinct hypoactive delirium and organ dysfunction could be a proper option in the therapeutic concept. A targeted therapy is available using the indirect parasympathomimetic physostigmine.

## References

[1] Marcantonio E. R. (2012). Postoperative delirium. *The Journal of the American Medical Association*.

[2] Hshieh T. T., Fong T. G., Marcantonio E. R., Inouye S. K. (2008). Cholinergic deficiency hypothesis in delirium: a synthesis of current evidence. *Journals of Gerontology Series A: Biological Sciences and Medical Sciences*.

[3] Nitsch R. M., Rossner S., Albrecht C. (1998). Muscarinic acetylcholine receptors activate the acetylcholinesterase gene promoter. *Journal of Physiology Paris*.

[4] Flacker J. M., Cummings V., Mach J. R., Bettin K., Kiely D. K., Wei J. (1998). The association of serum anticholinergic activity with delirium in elderly medical patients. *The American Journal of Geriatric Psychiatry*.

[5] Haase U., Rundshagen I. (2007). Pharmakotherapie—physostigmin post OP. *Anästhesiol Intensivmed Notfallmed Schmerzther*.

[6] Worek F., Mast U., Kiderlen D., Diepold C., Eyer P. (1999). Improved determination of acetylcholinesterase activity in human whole blood. *Clinica Chimica Acta*.

[7] Steiner L. A. (2011). Postoperative delirium. Part 1: pathophysiology and risk factors. *European Journal of Anaesthesiology*.

[8] Durand M. T., Becari C., de Oliveira M. (2014). Pyridostigmine restores cardiac autonomic balance after small myocardial infarction in mice. *PLoS ONE*.

[9] Delles C., Schmieder R. E. (2003). Endothelial function of the human renal vasculature. *Deutsche Medizinische Wochenschrift*.

[10] Borovikova L. V., Ivanova S., Zhang M. (2000). Vagus nerve stimulation attenuates the systemic inflammatory response to endotoxin. *Nature*.

